# Supplementary material for: Heat adaptation of phage T7 under an extended genetic code
Source: Virus Evol. 2021 Dec 1;7(2):veab100. doi: 10.1093/ve/veab100 (PMC8923235; doi:10.1093/ve/veab100)
Supplement: veab100_Supp [file veab100_supp.zip › Sequencing_Analysis.zip!/Sequencing_Analysis/S1/evidence/JC_SIDE_1_41.html]

BRESEQ :: Evidence


| New junction evidence | | | | | | | | | | | |
| --- | --- | --- | --- | --- | --- | --- | --- | --- | --- | --- | --- |
|  | | seq id | position | reads (cov) | reads (cov) | score | skew | freq | annotation | gene | product |
| \* | ? | NC\_001604.1 | 295 = | 5273 (1.260) | 27 (0.010) | 15/560 | 40.5 | 0.54% | intergenic (–/–) | *–/–* | –/– |
|  | ? | NC\_001604.1 | = 281 | 4936 (1.260) |  |  |  |  | intergenic (–/–) | *–/–* | –/– |
| Rejected: Coverage evenness skew score above cutoff. | | | | | | | | | | | |
| Rejected: Frequency below/above cutoff threshold. | | | | | | | | | | | |

Only 100 of 5296 total aligned reads displayed.  

`‑‑TCTCACAGTGTACGGACCTAAAGTTCCCCCATAGGGGGTACCTAAAGCCCAGCCAATCACCTAAAGTCAACCTTCGGTTGACCTTGAGGGTTCCCTAAGGGTTGGGGATGACCCTTGGGTTTGTCTTTGGGTGTTACCTTGAGTGTCTCTCTGTGTCCCTATCTGTTACAGTCTCCTAAAGTATCCTCCTAAAGTCACCTCCTAACGTCCATCCTAAAGCCAACACCTAAAGCCTACACCTAAAGACCCATCAAGTCAACGCCTATCTTAAAGTTTAAACATAAAGACCAGACCTAAAGACCAGACCTAAAGACACTACATAAAGACCAGACCTAAAGACGCCTTGTTGTTAGCCATAAAGTGATAACCTTTAATCATTGTCTTTATTAATACAACTCACTATAAGGAGAGACAACTTAAAGAGACTTAAAAGATTAATTTAAAATTTATCAAAAAGAGTATTGACTTAAAGTCTAACCTATAGGATACTTACAGCCATCGAGAGGGACACGGCGAATAGCCATCCCAATCGACACCGGGGTCAACCGGATAAGTAGACAGCCTGATAAGTCGCACGAAAAACAGGTATTGACAA  >  NC_001604.1/1‑595`  

                                                                                                                                                                                                                                                                                                        `|`                                                                                                                                                                                                                                                                                                              
`gggctcggagatgtgtataagagacagcccccATAGGGGGTACCTAAAGCCCAGCCAATCACCTAAAGTCAACCTTCGGTTGACCTTGAGGGTTCCCTAAGGGTTGGGGATGACCCTTGGGTTTGTCTTTGGGTGTTACCTTGAGTGTCTCTCTGTGTCCCTATCTGTTACAGTCTCCTAAAGTATCCTCCTAAAGTCACCTCCTAACGTCCATCCTAAAGCCAACACCTAAAGCCTACACCTAAAGACCCATCAAGTCAACGCCTATCTTAAAGTTTAAACAtaaagaccagacctaaag                                                                                                                                                                                                                                                                                                          <  1:575856/274‑1 (MQ=255)`  
   `ttttatggaacaggACCTAAATGTCCCCCCAAGGGGGTACCGAAAGCCCAGCCAAACCCCTAAAGTCAACCTTCGGTTGACCTTGAGGGTTCCCTAAGGGTTGGGGATGACCCTTGGGTTTTTCTTTGGGTGTTACCTTGAGTGTCTCTCTGTGTCCCTATCTGTTACAGTCTCCTAAAGTATCCTCCTAAAGTCACCTCCTAACGTCCATCCTAAAGCCAACACCTAAAGCCTACACCTAAAGACCCATCAAGTCAACGCCTATCTTAAAGTTTAAACAtaaagcccagacctaaagacc                                                                                                                                                                                                                                                                                                       <  1:70038/289‑1 (MQ=25)`  
   `agagacaggtACGGACCTATAGTTCCCCCATAGGGGGTACCTAAAGCCCAGCCAATCACCTAAAGTCAACCTTCGGTTGACCTTGAGGGTTCCCTAAGGGTTGGGGATGACCCTTGGGTTTGTCTTTGGGTGTTACCTTGAGTGTCTCTCTGTGTCCCTATCTGTTACAGTCTCCTAAAGTATCCTCCTAAAGTCACCTCCTAACGTCCATCCTAAAGCCAACACCTAAAGCCTACACCTAAAGACCCATCAAGTCAACGCCTATCTTAAAGTTTAAACAtaaagaccagacctaaagacc                                                                                                                                                                                                                                                                                                       <  1:321725/293‑1 (MQ=255)`  
   `agacaggtgtACGGACCTAAAGTTCCCCCATAGGGGGTACCTAAAGCCCAGCCAATCACCTAAAGTCAACCTTCGGTTGACCTTGAGGGTTCCCTAAGGGTTGGGGATGACCCTTGGGTTTGTCTTTGGGTGTTACCTTGAGTGTCTCTCTGTGTCCCTATCTGTTACAGTCTCCTAAAGTATCCTCCTAAAGTCACCTCCTAACGTCCATCCTAAAGCCAACACCTAAAGCCTACACCTAAAGACCCATCAAGTCAACGCCTATCTTAAAGTTTAAACAtaaagaccagacctaaagacc                                                                                                                                                                                                                                                                                                       <  1:100028/295‑1 (MQ=255)`  
         `gtgtACGGACCTAAAGTTCCCCCATAGGGGGTACCTAAAGCCCAGCCAATCACCTAAAGTCAACCTTCGGTTGACCTTGATGGTTCCCTAAGGGTTGGGTATGACCCTTGGGTTTGTCTTTGGGTGTTACCTTGAGTGTCTCTCTGTGTCCCTATCTGTTACAGTCTCCTAAAGTATCCTCCTAAAGTCACCTCCTAACGTCCATCCTAAAGCCAACACCTAAAGCCTCCACCTCAAGACCCATCAAGTCAACGCCTATCTTTAAGTTTCAACAtaaagaccagacctaactacacgccct                                                                                                                                                                                                                                                                                                 >  1:87954/1‑290 (MQ=255)`  
         `gtgtACGGACCTAAAGTTCCCCCATAGGGGGTACCTAAAGCCCAGCCAATCACCTAAAGTCAACCTTCGGTTGACCTTGAGGGTTCCCTGAGGGTTGGGGATGACCCTTGGGTTTGTCTTTGGGTGTTACCTTGAGTGTCTCTCTGTGTCCCTATCTGTTACAGTCTCCTAAAGTATCCTCCTAAAGTCACCTCCTAACGTCCATCCTAAAGCCAACACCTAAAGCCTACACCTAAAGACCCATCAAGTCAACGCCTATCTTAAAGTTTAAACATaaagaccaaacctaaagaccagacct                                                                                                                                                                                                                                                                                                 >  1:593433/1‑301 (MQ=255)`  
         `gtgtACGGACCTAAAGTTCCCCCATAGGGGGTACCTAAAGCCCAGCCAATCACCTAAAGTCAACCTTCGGTTGACCTTGAGGGTTCCCTAAGGGTTGGGGATGACCCTTGGGTTTGTCTTTGGGTGTTACCTTGAGTGTCTCTCTGTGTCCCTATCTGTTACAGTCTCCTAAAGTATCCTCCTAAAGTCACCTCCTAACGTCCATCCTAAAGCCAACACCTAAAGCCTACACCTAAAGACCCATCAAGTCAACGCCTATCTTAAAGTTTAAACAtaaagaccagacctaaagctgtctctt                                                                                                                                                                                                                                                                                                 >  1:207883/1‑292 (MQ=255)`  
         `gtgtACGGACCTAAAGTTCCCCCATAGGGGGTACCTAAAGCCCAGCCAATCACCTAAAGTCAACCTTCGGTTGACCTTGAGGGTTCCCTAAGGGTTGGGGATGACCCTTGGGTTTGTCTTTGGGTGTTACCTTGAGTGTCTCTCTGTGTCCCTATCTGTTACAGTCTCCTAAAGTATCCTCCTAAAGTCACCTCCTAACGTCCATCCTAAAGCCAACACCTAAAGCCTACACCTAAAGACCCATCAAGTCAACGCCTATCTTAAAGTTTAAAAATaaagaccagacctaaagaccagaact                                                                                                                                                                                                                                                                                                 >  1:219999/1‑301 (MQ=255)`  
         `gtgtACGGACCTAAAGTTCCCCCATAGGGGGTACCTAAAGCCCAGCCAATCACCTAAAGTCAACCTTCGGTTGACCTTGAGGGTTCCCTAAGGGTTGGGGATGACCCTTGGGTTTGTCTTCGGGTGTTACCTTGAGTCTCTCTCTGTGTCCCTATCTGTTACAGTCTCCTAAAGTATCCTCCTAAAGTCACCTCCTAACGTCCATCCTAAAGCCAACACCTAAAGCCTACACCTAAAGACCCATCAAGTCAACGCCTATCTTAAAGTTTAAACATaaagaccagacctaaagaccagacct                                                                                                                                                                                                                                                                                                 >  1:529466/1‑301 (MQ=255)`  
         `gtgtACGGACCTAAAGTTCCCCCATAGGGGGTACCTAAAGCCCAGCCAATCACCT‑AAGTCAACCTTCGGTTGACCTTGAGGGTTCCCTAAGGGTTGGGGATGACCCTTGGGTTTGTCTTTGGGTGTTACCTTGAGTGTCTCTCTGTGTCCCTATCTGTTACAGTCTCCTAAAGTATCCTCCTAAAGTCACCTCCTAACGTCCATCCTAAAGCCAACACCTAAAGCCTACACCTAAAGACCCATCAAGTCAACGCCTATCTTAAAGTTTAAACAtaaagaccagacctaaagacctgtctct                                                                                                                                                                                                                                                                                                >  1:370856/1‑294 (MQ=255)`  
           `gtACGGACCTAAAGTTCCCCCATAGGGGGTACCTAAAGCCCAGCCAATCACCTAAAGTCAACCTTCGGTTGACCTTGAGGGTTCCCTAAGGGTTGGGGATGACCCTTGGGTTTGTCTTTGGGTGTTACCTTGAGTGTCTCTCTGTGTCCCTATCTGTTACAGTCTCCTAAAGTATCCTCCTAAAGTCACCTCCTAACGTCCATCCTAAAGCCAACACCTAAAGCCTACACCTACAGACCCATCAAGTCAACGCCTATCTTAAAGTTTAAACAtaaagaccagacctaaagaccctgtctct                                                                                                                                                                                                                                                                                               >  1:249840/1‑293 (MQ=255)`  
           `gtACGGACCTAAAGTTCCCCCATAGGGGGTACCTAAAGCCCAGCCAATCACCTAAAGTCAACCTTCGGTTGACCTTGAGGGTTCCCTAAGGGTTGGGGATGACCCTTGGGTTTGTCTTTGGGTGTTACCTTGAGTGTCTCTCTGTGTCCCTATCTGTTACAGTCTCCTAAAGTATCCTCCTAAAGTCACCTCCTAACGTCCATCCTAAAGCCAACACCTAAAGCCTACACCTAAAGACCCATCAAGTCAACGCCTATCTTAAAGTTTAAACAtaaagaccagacctaaagctgtctcttat                                                                                                                                                                                                                                                                                               >  1:453996/1‑290 (MQ=255)`  
           `gtACGGACCTAAAGTTCCCCCATAGGGGGTACCTAAAGCCCAGCCAATCACCTAAAGTCAACCTTCGGTTGACCTTGAGGGTTCCCTAAGGGTTGGGGATGACCCTTGGGTTTGTCTTTGGGTGTTACCTTGAGTGTCTCTCTGTGTCCCTATCTGTTACAGTCTCCTAAAGTATCCTCCTAAAGTCACCTCCTAACGTCCATCCTAAAGCCAACACCTAAAGCCTACACCTAAAGACCCATCAAGTCAACGCCTATCTTAAAGTTTAAACATAAagaccagacctaaagaccagacataa                                                                                                                                                                                                                                                                                               >  1:518933/1‑301 (MQ=255)`  
               `ggACCTAAATTTCCCCCATATGGGGTACCTAAAGCCCAGCCAATCACCTAAAGTCAACCTTCGGTTGATCTTGAGGGTTCCCTAAGGGTTGGGGATGACCCTTGGGTTTGTCTTTGGGTGTTACCTTGAGTGTCTCTCTGTGTCCCTATCTGTTACAGTCTCCTAAAGTATCCTCCTAAAGTCACCTCCTAACGTCCATCCTAAAGCCAACACCTAAAGCCTACACCTAAAGACCCATCAAGTCAACGCCTATCTTAAAGTTTAAACATAAAGACcagacctaaagaccagacctaaagac                                                                                                                                                                                                                                                                                           <  1:668550/301‑1 (MQ=255)`  
                `gACCTAAAGTTCCCCCATAGGGGGTACCTAAAGCCCAGCCAATCACCTAAATTCAACCTTCGGTTGACCTTGAGGGTTCCCTACGGGTTGGGGATGACCCTTGGGTTTGTCTTTGGCTGTTACCTTGAGTGCCGCTCTGTGTCCCTATCTGTTACAGTCTCCTACAGTCTCCTCCTAAAGTCACCTCCTAACGTCCATCCTAAAGCCAACCCCTAAAGCCTACACCTAAAGACCCATCTAGTCCACGCCTATCTTAAAGATTAACCATAAAgaccaaacctaaacaccaaacctgaactct                                                                                                                                                                                                                                                                                          >  1:653287/1‑297 (MQ=25)`  
                `gACCTAAAGTTCCCCCATAGGGGGTACCTAAAGCCCAGCCAATCACCTAAAGTCAACCTTCGGTTGACCTTGAGGGTTCCCTAAGGGTTGGGGATGACCCTTGGGTTTGTCTTTGGGTGTTACCTTGAGTGTCTCTCTGTGTCCCTATCTGTTACAGTCTCCTAAAGTATCCTCCTAAAGTCACCTCCTAACGTCCATCCTAAAGCCAACACCTAAAGCCTACACCTAAAGACCCATCAAGTCAACGCCTATCTTAAAGTTTAAACATAAAGACCAGACCTAAAGACCAGACCTAAAGaca                                                                                                                                                                                                                                                                                          >  1:243013/1‑301 (MQ=255)`  
                `gACCTAAAGTTCCCCCATAGGGGGTACCTAAAGCCCAGCCAATCACCTAAAGTCAACCTTCGGTTGACCTTGAGGGTTCCCTAAGGGTTGGGGATGACCCTTGGGTTTGTCTTTGGGTGTTACCTTGAGTGTCTCTCTGTGTCCCTATCTGTTACAGTCTCCTAAAGTATCCTCCTAAAGTCACCTCCTAACGTCCATCCTAAAGCCAACACCTAAAGCCTACACCTAAAGACCCATCAAGTCAACGCCTATCTTAAAGTTTAAACATAAAGACCAGACCTAAAGACCAGACCTAAAGaca                                                                                                                                                                                                                                                                                          >  1:429784/1‑301 (MQ=255)`  
                `gACCTAAAGTTCCCCCATAGGGGGTACCTAAAGCCCAGCCAATCACCTAAAGCCAACCTTCGGTTGACCTTGAGGGTTCCCTAAGGGTTGGGGATGACCCTTGGGTTTGTCTTTGGGTGTTACCTTGAGTGTCTCTCTGTGTCCCTATCTGTTACAGTCTCCTAAAGTATCCTCCTAAAGTCACCTCCTAACGTCCATCCTAAAGCCAACACCTAAAGCCTACACCTAAAGACCCATCAAGTCAACGCCTATCTTAAAGTTTAAACATAAAGACCAGACCTAAAGACCAGACCTAAAGaca                                                                                                                                                                                                                                                                                          >  1:599757/1‑301 (MQ=255)`  
                 `aCCTAAAGTTCCCCCATAGGGGGTACCTAAAGCCCAGCCAATCACCTAAAGTCAACCTTCTGTTGACCTTGAGGGTTCCCTAAGGGTTGGGGATGACCCTTGGGTTTGTCTTTGGGTGTTACCTTGAGTGTCTCTCTGTGTCCCTATCTGTTACAGTCTCCTAAAGTATCCTCCTAAAGTCACCTCCTAACGTCCATCCTAAAGCCAACACCTAAAGCCTACACCTAAAGACCCATCAAGTCAACGCCTATCTTAAAGTTTAAACATAAAGACCAGACCTAAAGACCAGACCTAAAGacac                                                                                                                                                                                                                                                                                         <  1:382524/301‑1 (MQ=255)`  
                       `aGTTCCCCCATAGGAGGTACCTAAAGCCCAGCCAATCACCTAAAGTCAACCTTCGGTTGACCTTGAGGGTTCCCTAAGGGTTGGGGATGACCCTTGGGTTTGTCTTTGGGTGTTACCTTGAGTGTCTCTCTGTGTCCCTATCTGTTACAGTCTCCTAAAGTATCCTCCTAAAGTCACCTCCTAACGTCCATCCTAAAGCCAACACCTAAAGCCTACACCTAAAGACCCATCAAGTCAACGCCTATCTTAAAGTTTAAACATAAAGACCAGACCTAAAGACCAGCCCTAAAGACACTACATa                                                                                                                                                                                                                                                                                   >  1:261635/1‑301 (MQ=255)`  
                        `gTTCCCCCATAGGGGGTACCTAAAGCCCAGCCAATCACCTAAAGTCAACCTTCGGTTGACCTTGAGGGTTCCCTAAGGGTTGGGGATGACCCTTGGGTTTGTCTTTGGGTGTTACCTTGAGTGTCTCTCTGTGTCCCTATCTGTTACAGTCTCCTAAAGTATCCTCCTAAAGTCACCTCCTAACGTCCATCCTAAAGCCAACACCTAAAGCCTACACCTAAAGACCCCTCAAGTCAACGCCTATCTTAAAGTTTAAACATAAAGACCAAACCTACAGACCAGACCTAAAGAAACTACATaa                                                                                                                                                                                                                                                                                  >  1:124282/1‑301 (MQ=255)`  
                           `ccccccTGGGGGGTACCTAAAGCCCATCCTATCACCTAAAGTCAACCTTCGGTTGACCTTGAGGGTTCCCTAAGGGTTGGTGATGACCCTTGGGTTTGTCTTTGGGTGTTACCTTGAGTGTCTCTCTGTGTCCCTATCTGTTACAGTCTCCTAAAGTATCCTCCTAAAGTCACCTCCTAACGTCCATCCTAAAGCCAACACCTAAAGCCTACACCTAAAGACCCATCAAGTCAACGCCTATCTTAAAGTTTAAACATAAAGACCAGACCTAAAGACCAGACCTAAAGACACTACATAAaga                                                                                                                                                                                                                                                                               <  1:141993/301‑1 (MQ=255)`  
                           `cccccATAGGGGGTACCTAAAGCCCAGCCAATCACCTAAAGTCAACCTTCGGTTGACCTTTAGGGTTCCCTAAGGGTTGGGGATGACCCTTGGGTTTGTCTTTGGGTGTTACCTTGAGTGTCTCTCTGTGTCCCTATCTGTTACAGTCTCCTAAAGTATCCTCCTAAAGTCACCTCCTAACGTCCATCCTAAAGCCAACACCTAAAGCCTACACCTAAAGACCCATCAAGTCAACGCCTATCTTAAAGTTTAAACATAAAGACCAGACCTAAAGACCAAACCTAAAGACACTACATAAaga                                                                                                                                                                                                                                                                               >  1:434951/1‑301 (MQ=255)`  
                            `ccccATAGGGGGTACCTAAAGCCCAGCCAATCACCTAAATTCAACCTTCGGTTGACCTTGAGGGTTCCCTAAGGGTTGGGGATGACCCTTGGGTTTGTCTTTGGGTGTTACCTTGAGTGTCTCTCTGTGTCCCTATCTGTTACAGTCTCCTAAAGTATCCTCCTAAAGTCACCTCCTAACGTCCATCCTAAAGCCAACACCTAAAGCCTACACCTAAAGACCCATCAAGTCAACGCCTATCTTAAAGTTTAAACATAAAGACCAGACCTAAAGACCAGACCTAAAGACACTACATAAagac                                                                                                                                                                                                                                                                              <  1:176993/301‑1 (MQ=255)`  
                            `ccccATAGGGGGTACCTAAAGCCCAGCCAATCACCTAAAGTCAACCTTCGGTTGACCTTGAGGGTTCCCTAAGGGTTGGGGATGACCCTTGGGTTTGTCTTTGGGTGTTACCTTGAGTGTCTCTCTGTGTCCCTATCTGTTACAGTCTCCTAAATTATCCTCCTAAAGTCACCTCCTAACGTCCATCCTAAAGCCAACACCTAAAGCCTACACCTAAAGACCCATCAAGTCAACGCCTATCTTAAAGTTTAAACATAAAGACCAGACCTAAAGACCAGACCTAAAGACACTACATAAagac                                                                                                                                                                                                                                                                              <  1:213449/301‑1 (MQ=255)`  
                            `ccccATAGGGGGTACCTAAAGCCCAGCCAATCACCTAAAGTCAACCTTCGGTTGACCTTGAGGGTTCCCTAAGGGTTGGGGATGACCCTTGGGTTTGTCTTTGGGTGTTACCTTGAGTGTCTCTCTGTGTCCCTATCTGTTACAGTCTCCTAAAGTATCCTCCTAAAGTCACCTCCTAACGTCCATCCTAAAGCCAACACCTACAGCCTACACCTAAAGACCCATCAAGTCAACGCCTATCTTAAAGTTTAAACATAAAGACCAGACCTAAAGACCAGACCTAAAGACACTACATAAagaa                                                                                                                                                                                                                                                                              >  1:184000/1‑300 (MQ=255)`  
                             `agacaggggggTACCTAAAGCCCAGCCAATCACCTAAAGTCAACCTTCGGTTGACCTTGAGGGTTCCCTAAGGGTTGGGGATGACCCTTGGGTTTGTCTTTGGGTGTTACCTTGAGTGTCTCTCTGTGTCCCTATCTGTTACAGTCTCCTAAAGTATCCTCCTAAAGTCACCTCCTAACGTCCATCCTAAAGCCAACACCTAAAGCCTACACCTAAAGACCCATCAAGTCAACGCCTATCTTAAAGTTTAAACATAAAGACCAGACCTAAAGACCAGACCTAAAGACACTACATAAagacc                                                                                                                                                                                                                                                                             <  1:327854/295‑1 (MQ=255)`  
                               `cATAGGGGGTACCTAAAGCCCAGCCAATCACCTAAAGTCAACCTTCGGTTGACCTTGAGGGTTCCCTAAGGGTTGGGGATGACCCTTGGGTTTGTCTTTGGGTGTTACCTTGAGTGTCTCTCTGTGTCCCTATCTGTTACAGTCTCCTAAAGTATCCTCCTAAAGTCACCTCCTAACGTCCATCCTAAAGCCAACACCTAAAGCCTACACCTAAAGACCCATCAAGTCAACGCCTATCTTAAAGTTTAAACATAAAGACCAGACCTAAAGACCAGACCTAAAGACACTACATAAagaccaa                                                                                                                                                                                                                                                                           >  1:477492/1‑300 (MQ=255)`  
                                          `ccTAAAGCCCAGCCAATCACCTAAAGTCAACCTTCGGTTGACCTTGAGGGTTCCCTAAGGGTTGGGGATGACCCTTGGGTTTGTCTTTGGGTGTTACCTTGAGTGTCTCTCTGTGTCCCTATCTGTTACAGTCTCCTAAAGTATCCTCCTAAAGTCACCTCCTAACGTCCATCCTAAAGCCAACACCTAAAGCCTACACCTAAAGACCCATCAAGTCAACGCCTATCTTAACGTTTAAACATAAAGACcagacctaaagaccagacctaaagacctgtctcttatacacatctccgagccc                                                                                                                                                                                                                                                                >  1:581949/1‑274 (MQ=255)`  
                                                            `aCCTAAAGTCAACCTTCGGTTGACCTTGAGGGTTCCCTAAGGGTTGGGGATGACCCTTGGGTTTGTCTTTGGGTGTTACCTTGAGTGTCTCTCTGTGTCCCTATCTGTTACAGTCTCCTAAAGTATCCTCCTAAAGTCACCTCCTAACGTCCATCCTAAAGCCAACACCTAAAGCCTACACCTAACGACCCATCAAGTCAACGCCTATCTTAAAGTTTAAACATAAAGACCAGACCTAAAGACCATACCTAAAGACACTACATAAAGACCAGCCCTAAAGACGCCTTGTTGTTAGCCATaa                                                                                                                                                                                                                                              >  1:465725/1‑301 (MQ=255)`  
                                                             `ccTAAAGTCAACCTTCGGTTGACCTTGAGGGTTCCCTAAGGGTTGGGGATGACCCTTGGGTTTGTCTTTGGGTGTTACCTTGAGTGTCTCTCTGTGTCCCTATCTGTTACAGTCTCCTAAAGTATCCTCCTAAAGTCACCTCCTCACGTCCATCCTAAAGCCAACACCTAAAGCCTACACCTAAAGACCCATCAAGTCAACGCCTATCTTAAAGTTTAAACATAAAGACCAGACCTAAAGACCAGACCTAAAGACACTACATAAAGACAAGACCTAAAGActgtcacctaaacacatctcc                                                                                                                                                                                                                                             >  1:558583/1‑281 (MQ=255)`  
                                                              `cTAAAGTCAACCTTCGGTTGACCTTGAGGGTTCCCTAAGGGTTGGGGATGACCCTTGGGTTTGTCTTTGGGTGTTACCTTGAGTGTCTCTCTGTGTCCCTATCTGTTACAGTCTCCTAAAGTATCCTCCTAAAGTCACCTCCTAACGTCCATCCTAAAGCCAACACCTAAAGCCTACACCTAAAGACCCATCAAGTCAACGCCTATCTTAAAGTTTAAACATAAAGACCAGACCTAAAGACCAGACCTAAAGACACTACATAAAGACCAGACCTAAAGACGCCTTGTTGTTAGCCATAAAg                                                                                                                                                                                                                                            <  1:190281/301‑1 (MQ=255)`  
                                                                      `aaCCGTCGGTTGACCTTGAGTGTTCCCTAAGGGCTGGGGATGACCCTTGGGTTTGTCTTTGGGTGTGACCTTGAGTGTCTCTCTGTGTCCCATTCTGTTACAGTCTCCTAAAGTATCCTCCTAAAGTCACCTCCTAACGTCCATCCTAAAGCCAACACCTAAAGCCTACACCTAAAGACCCATCAAGTCAACGCCTATCTTAAAGTTTAAACATAAAGACCAGACCTAAAGACCAGACCTAAAGACACTACATAAAGACCAGACCTAAAGACGCCTTGTTGTTAGCCATAAAGTGATAAcc                                                                                                                                                                                                                                    <  1:491377/301‑1 (MQ=255)`  
                                                                       `aCCTTCGGTTGACCTTGAGGGTTCCCTAAGGGTTGGGGATGACCCTTGGGTTTGTCTTTGGGTGTTACCTTGAGTGTCTCTCTGTGTCCCTATCTGTTACAGTCTCCTAAAGTATCCTCCTAAAGTCACCTCCTAACGTCCATCCTAAAGCCAACACCTAAAGCCTACACCTAAAGACCCATCAAGTCAACGCCTATCTTAAAGTTTAAACATAAAGTCCAGACCTAAAGACCAGACCTAAAGACACTACATAAAGACCAGACCTAAAGACGCCTTGTTGTTAGCCATAAAGTGATAAccc                                                                                                                                                                                                                                   >  1:111015/1‑300 (MQ=255)`  
                                                                       `aCCTTCGGTTGACCTTGAGGGTTCCCTAAGGGTTGGGGATGACCCTTGGGTTTGTCTTTGGGTGTTACCTTGAGTGTCTCTCTGTGTCCCTATCTGTTACAGTCTCCTAAAGTATCCTCCTAAAGTCACCTCCTAACGTCCATCCTAAAGCCAACACCTAAAGCCTACACCTAAAGACCCATCAAGTCAAAGCCTATCTTAAAGTTTAAACATAAAGACCAGACCTAAAGACCAGACCTAAAGACACTACATAAAGACCAGACCTAAACACGCCTTGTTGTTAGCCATAAAGTGATAAccc                                                                                                                                                                                                                                   >  1:202064/1‑300 (MQ=255)`  
                                                                             `ggTTTACCCTGAGGGTTCCCTTAGGGTTTGGGATGACCCTTTGGTTTGTCTTTGGGTGTTACCTTGAGTGTCTCTCTGTGTCCCTATCTGTTACAGTCTCCTAAAGTATCCTCCTAAAGTCACCTCCTAACGTCCATCCTAAAGCCAACACCTAAAGCCTACACCTAAAGACCCATCAAGTCAACGCCTATCTTAAAGTTTAAACATAAAGACCAGACCTAAAGACCAGACCTAAAGACACTACATAAAGACCAGACCTAAAGACGCCTTGTTGTTAGCCATAAAGTGATAACCTTTAATc                                                                                                                                                                                                                             <  1:606681/301‑1 (MQ=255)`  
                                                                             `ggTTGACCTTGAGGGTTCCCTAAGGGTTGGGGATGACCCTTGGGTTTGTTTTTGGGTGTTACCTTGAGTGTCTCTCTGTGTCCCTATCTGTTACAGTCTCCTAAAGTATCCTCCTAAAGTCACCTCCTAACGTCCAACCTAAAGCCAACACCTAAAGCCTACATCTAAAGACCCATCAAGTCAACGCCTATCTTAAAGTTTAAACATAAAGACCAGACCTAAAGACCAGACCTAAAGACACTACATAAAGACCAGACCTAAAGACGCCTTGTTGTTAGCCATAAAGTGATAACCTTTAATc                                                                                                                                                                                                                             <  1:296607/301‑1 (MQ=255)`  
                                                                             `ggTTGACCTTGAGGGTTCCCTAAGGGTTGGGGATGACCCTTGGGTTTGTCTTTGGGTGTTACCTTGAGTGTCTCTCTGTGTCCCTATCTGTTACAGTCTCCTAAAGTATCCTCCTAAAGTCACCTCCTAACGTCCATCCTAAAGCCAACACCTAAAGCCTACACCTAAAGACCCATCAAGTCAACGCCTATCTTAAAGTTTAAACATAAAGACCAGACCTAAAGACCAGACCTAAAGACACTACATAAAGACCAGACCTAAAGACGCCTTGTTGTTAGCCATAAAGTGATAACCTTTAATc                                                                                                                                                                                                                             <  1:570857/301‑1 (MQ=255)`  
                                                                             `ggTTGACCTTGAGGGTTCCCTAAGGGTTGGGGATGAACCTTGGTTTAGTCTTTGGGTGTTACCTTGAGTGTCTCTCTGTGTCCCTATCTGTTACAGTCTCCTAAAGTATCCTCCTAAAATCACCTCCTAACGTCCATCCTAAAGCCAACACCTAAAGCCTACACCTAAAGACGCATCAAGTCAACGCCTATCTTAAAGTTTAAACATAAAGACCAGACCTAAAGACCAGACCTAAAGACACTACATAAAGACCAGACCTAAAGACGCCTTGTTGTTAGCCATAAAGTGATAACCTTTAATc                                                                                                                                                                                                                             <  1:93687/301‑1 (MQ=255)`  
                                                                                 `gACCTTGAGGGTTCCCTAAGGGTTGGGGATGACCCTTTGGTTTGTCTTTGGGTGTTACCTTGAGTGTCTCTCTGTGTCCCTATCTGTTACAGTCTCCTAAAGTATCCTCCTAAAGTCACCTCCTAACGTCCATCCTAAAGCCAACACCTAAAGCCTACACCTAAAGACCCATCAAGTCAACGCCTATCTTAAAGTTTAAACATAAAGACCAGACCTAAAGACCAGACCTAAAGACACTACATAAAGACCAGACCTAAAGACGCCTTGTTGTTAGCCATAAAGTGATAACCTTTAATCAttt                                                                                                                                                                                                                         >  1:117322/1‑300 (MQ=255)`  
                                                                                   `ccTTGAGGGTTCCCTAAGGGTTGGGGATGACCCTTGGGTTTGTCTTTGGGTGTTACCTTGAGTGTCTCTCTGTGTCCCTATCTGTTACAGTCTCCTAAAGTATCCTCCTAAAGTCACCTCCTAACGTCCATCCTAAAGCCAACACCTAAAGCCTACACCTAAAGACCCATCAAGTCAACGCCTATCTTAAAGTTTAAACATAAAGACCAGACCTAAAGACCAGACCTAAAGACACTACATAAAGACCAGACCTAAAGACGCCTTGTTGTTAGCCATAAAGTGATAACCTTTAATcctgtct                                                                                                                                                                                                                       >  1:613184/1‑295 (MQ=255)`  
                                                                                         `gggTTCCCTAAGGGTTGGGGATGACCCTTGGGTTTGTCTTTGGGTGTTACCTTGAGTGTCTCTCTGTGTCCCTATCTGTTACAGTCTCCTAAAGTATCCTCCTAAAGTCACATCCTAACGTACATCCTAAAGCCAACACCTAAAGCCTACACCTAAAGACCCATCAAGTCAACGCCTATCTTAAAGTTTAAACATAAAGACCAGACCTAAAGACCAGACCTAAAGACACTACATAAAGACCAGACCTAAAGACGCCTTGTTGTTAGCCATAAAGTGATAACCTTTAATCATTGTCTttatt                                                                                                                                                                                                                 <  1:485103/301‑1 (MQ=255)`  
                                                                                         `gggTTCCCTAAGGGTTGGGGATGACCCTTGGGTTTGTCTGTGGGTGTTACCTTGAGTGTCTCTCTGTGTCCCTATCTGTTACAGTCTACTAAAGTATCCTCCTAAAGTCACCTCCTAACGTCAATCATAAAGCCAACACCTAAAGCCTACACCTAAAGACCCATCAAGTCAACGCCTATCTTAAAGTTTAAACATAAAGACCAGACCTAAAGACCAGACCTAAAGACACTACATAAAGACCAGACCTAAAGACGCCTTGTTGTTAGCCATAAAGTGATAACCTTTAATCATTGTCTttatt                                                                                                                                                                                                                 <  1:351609/301‑1 (MQ=255)`  
                                                                                              `cccTAAGGGTTGGGTATGACCCTTTGGTTTGTCTTTGGGTGTTACCTTGAGTGTCTCTCTGTTGCCCCATCTGTTACAGTCTCCTAAAGTATCCTCCTAAAGTAACCTCCCAACGTCCATCCTAAAGCCAACACCTAAAGCCTACATCTAAAGACCCATCAAGTCAACGCCTATCTTAAAGTTTAAACATAAAGACCAGACCTAAAGACCAGACCTAAAGACACTACATAAAGACCAGACCTAAAGACGCCTTGTTGTTAGCCATAAAGTGATAACCTTTAATCATTGTCTTTATTAATAc                                                                                                                                                                                                            <  1:302845/301‑1 (MQ=255)`  
                                                                                               `ccTAAGGGTTGGGGATGACCCTTGGGTTTGTCTTTGGGTGTTACCTTGAGTGTCTCTCTGTGTCCCTATCTGTTACAGTCTCCTAAAGTATCCTCCTAACGTCACCTCCTAACGTCCATCCTAAAGCCAACACCTAAAGCCTACACCTAAAGACCCATCAAGTCAACGCCTATCTTAAAGTTTAAACATAAAGACCAGACCTAAAGACCAGACCTAAAGACACTACATAAAGACCAGACCTAAAGACGCCTTGTTGTTAGCCATAAAGTGATAACCTTTAATCATTGTCTTTATTAATACa                                                                                                                                                                                                           >  1:2559/1‑301 (MQ=255)`  
                                                                                                 `tAAGGGTTGGGGATGCCCCTTGGGTTTGTCTGTGGGTGTGACGTTGAGTGTCTCTCTGTGTCCCTATCACTTACAGTCTCCTAAAGTATCCTCCTAAAGTCACCCCCTAACGTCCATCCTAAAGCCAACACCTAAAGCCTACACCTAAAGACCCATCAAGTCAACGCCTATCTTAAAGTTTAAACATAAAGACCAGACCTAAAGACCAGACCTAAAGACACTACATAAAGACCAGACCTAAAGACGCCTTGTTGTTAGCCATAAAGTGATAACCTTTAATCATTGTCTTTATTAATACAAc                                                                                                                                                                                                         <  1:136554/301‑1 (MQ=255)`  
                                                                                                   `aGGGTTGGGGATGACCCTTTGGTTTGTCTTTGGGTGTTACCTTGAGTGTCTCTCTGTGTCCCTATCTGTTACAGTCTCCTAAAGTATCCTCCTAAAGTCACCTCCTAACGTCCATCCTAAAGCCAACACCTAAAGCCTACACCTAAAGACCCATCAAGTCAACGCCTATCTTAAAGTTTAAACATAAAGACCAGACCTAAAGACCAGACCTAAAGACACTACATAAAGACCAGACCTAAAGACGCCTTGTTGTTAGCCATAAAGTGATAACCTTTAATCATTGTCTTTATTAATACAACTc                                                                                                                                                                                                       <  1:400258/301‑1 (MQ=255)`  
                                                                                                        `tgacagcgtctcTTGGTGTTTTCTTTTGGTTTTACCTTGAGTGTCTTTCTGTGTCCCTATCTGTTACAGTCTCCTAAACTATCCTTCTAAAGTCACCTCCTAACGTCCATCCTAAAGCCAACACCTAAAGCCTACACCTAAAGACCCATCAAGTTAACGCCTATCTTAAAGTTTAAACATAAAGACCAGACCTAAAGACCAGACCTAAAGACACTACATAAATACCAGACCTAAAGACGCCTTGTTGTTAGCCATAAAGTGATATCCTTTAATCATTGTCTTTATTAATACAACTCACtat                                                                                                                                                                                                  <  1:315892/290‑1 (MQ=255)`  
                                                                                                           `ggATGACCCTTGGGTTTGTCTTTGGGTGTTACCTTGAGTGTCTCTCTGTGTCCCTATCTGTTACAGTCTCCTAAAGTATCCTCCTAAAGTCACCTCCTAACGTCCATCCTAAAGCCAACACCTAAAGCCTACACCTACAGACCCATCAAGTCAACTCCTATCTTAAAGTTTAAACATAAAGACCAGACCTAAAGACCAGACCTAAAGACACTACATAAAGACCAGACCTAAAGACGCCTTGTTGTTAGCCATAAAGTGATAACCTTTAATCACTGTCtcttatacacatctccgaacccac                                                                                                                                                                                               >  1:291077/1‑278 (MQ=255)`  
                                                                                                            `tgtgtataagagacaggTCTTTGGGTGTTAACTCGAGTGTCTCTCTGTGTACCTATCTATTACAGTCTCCTAAAGTAACCTCCTAAAGTCACCTCCTAACGTCCATCCTAAATACAACACCTAAAGCCTACACCTAAAGACCCATCAAGTCAACGCCTATCTTAAAGTTTAAACATAAAGACCAGACCTAAAGACCAGACCTAAAGACACTACATAAAGACCAGACCTAAAGACGCCTTGTTGTTAGCCATAAAGTGATAACCTTTAATCATTGTCTTTATTAATACAACTCACTATAAgg                                                                                                                                                                                              <  1:661992/285‑1 (MQ=255)`  
                                                                                                            `gATGACCCTTGGGTTTGTCTTTGGGTGTTACCTTGAGTGTCTCTCTGTGTCCCTATCTGTTACAGTCTCCTAAAGTATCCTCCTAAAGTCACCTCCTAACGTCCATCCTAAAGCCAACACCTAAAGCCTACACCTAAAGACCCATCAAGTCAACGCCTATCTTAAAGTTTAAACATAAAGATCAGACCTAAAGACCAGACCTAAAGACACTACATAAAGACCAGACCTAAAGACGCCTTGTTGTTAGCCATAAAGTGATAACCTTTAATCATTGTCTTTATTAATACAACTCACTATAAgg                                                                                                                                                                                              <  1:471817/301‑1 (MQ=255)`  
                                                                                                             `agagacagtGGGTTTGTCTTTGGGTGTTACCTTGAGTGTCTCTCTGTGTCCCTATCTGTTACAGTCTCCTAAAGTATCCTCCTAAAGTCACCTCCTAACGTCCATCCTAAAGCCAACACCTAAAGCCTACACCTAAAGACCCATCAAGTCAACGCCTATCTTAAAGTTTAAACATAAAGACCAGACCTAAAGACCAGACCTAAAGACACTACATAAAGACCAGACCTAAAGACGCCTTGTTGTTAGCCATAAAGTGATAACCTTTAATCATTGTCTTTATTAATACAACTCACTATAAGga                                                                                                                                                                                             <  1:195674/293‑1 (MQ=255)`  
                                                                                                                 `cccTTGGGTTTGTCTTTGGGTGTTACCTTGAGTGTCTCTCTGTGTCCCTATCTGTTACAGTCTCCTAAAGTATCCTCCTAAAGTCACCTCCTAACGTCCATCCTAAAGCCAACACCTAAAGCCTACACCTAAAGACCCATCAAGTCAACGCCTATCTTAAAGTTTAAACATAAAGACCAGACCTAAAGACCAGACCTAAAGACACTACATAAAGACCAGACCTAAAGACGACTTGTTGTTAGCCATAAAGTGATAACCTTTAATCATTGTCTTTATTAATACAACTCCCTATCAGgactgt                                                                                                                                                                                         >  1:340329/1‑297 (MQ=255)`  
                                                                                                                     `gaggTATGTCTTGGGGTGTTACCTGGAGTGACTCTCTATGTCCGTATCTGTTACAGTCACCTAAAATATCCACCTAAAGACACCGCCTAACGTCCAACCTAAGGACAACGCCTAAAGCCTAAGACTAAAGATCCATAAAGTCAACACCTATCATAAAGTTTAAACATAAAGACCAGACCTAAAGACCAGACCTAAAGACACTACAGAAAGACCAGACCTAAAGACGCCTTGTTGTTAGCCATAAAGTGATAACCTTTAATCATTGTCTTTATTAATACAACTCACTATAAGGAGAGACAAc                                                                                                                                                                                     <  1:334010/299‑1 (MQ=255)`  
                                                                                                                           `cagcTTTGGGTGTTACCTTGAGTGTCTCTCTGTGTCCCTATCTGTTACAGTCTCCTAAAGTATCCTCCTAAAGTCACCTCCTAACGTCCATCCTAAAGCCAACACCTAAAGCCTACACCTAAAGACCCATCAAGTCAACGCCTATCTTAAAGTTTAAACATAAAGACCAGACCTAAAGACCAGACCTAAAGACACTACATAAAGACCAGACCTAAAGACGCCTTGTTGTTAGCCATAAAGTGATAACCTTTAATCATTGTCTTTATTAATACAACTCACTATAAGGAGAGACAACTTAAag                                                                                                                                                                               <  1:28635/298‑1 (MQ=255)`  
                                                                                                                           `aagagacaggTGTTACCTTGAGTGTCTCTCTGTGTCCCTATCTGTTACAGTCTCCTAAAGTATCCTCCTAAAGTCACCTCCTAACGTCCATCCTAAAGCCAACACCTAAAGCCTACACCTAAAGACCCATCAAGTCAACGCCTATCTTAAAGTTTAAACATAAAGACCAGACCTAAAGACCAGACCTAAAGACACTACATAAAGACCAGACCTAAAGACGCCTTGTTGTTAGCCATAAAGTGATAACCTTTAATCATTGTCTTTATTAATACAACTCACTATAAGGAGAGACAACTTAAag                                                                                                                                                                               <  1:552686/293‑1 (MQ=255)`  
                                                                                                                            `gTCTTTGGGTGTTACCTTGAGTGTCTCTCTGTGTCCCTATCTGTTACAGTCTCCTAAAGTATCCTCCTAAAGTCACCTCCTAACGTCCATCCTAAAGCCAACACCTAAAGCCTACACCTAAAGACCCATCAAGTCAACGCCTATCTTAAAGTTTAAACATAAAGACCAGACCTAAAGACCAGACCTAAAGACACTACATAAAGACCAGACCTAAAGACGCCTTGTTGTTAGCCATAAAGTGATAACCTTTAATCATTGTCTTTATTAATACAACTCACTATAAGGAGAGACAACTTAAaga                                                                                                                                                                              >  1:154842/1‑301 (MQ=255)`  
                                                                                                                                  `gggTGTTACCTTGAGTGTCTCTCTGTGTCCCTATCTGTTACAGTCTCCTAAAGTATCCTCCTAAAGTCACCTCCTAACGTCCATCCTAAAGCCAACACCTAAAGCCTACACCTAAAGACCCATCAAGTCAACGCCTATCTTAAAGTTTAAACATAAAGACCAGACCTAAAGACCAGACCTAAAGACACTACATAAAGACCAGACCTAAAGACGCCTTGTTGTTAGCCATAAAGTGATAACCTTTAATCATTGTCTTTATTAATACAACTCACTATAAGGAGAGACAACTTAAAGAGACTTa                                                                                                                                                                        >  1:523243/1‑301 (MQ=255)`  
                                                                                                                                      `tttACTTTGCGTGTCTCTCGTATTCCCTATATGTTACAGCATCCTCAACTATCCTACTTCACGCACCTCCTAACGTCCATCTTTAAGCCACCACCTAAAGCCTACACCTAAAGAACCATCAAGTCAACGCCTATATTAAAGTTTAAACATAAATACCAGCACTAAAGTCCAGACCTAAAGACACTACATAAAGACCAGACCTAAAGACGCCTTGTTGTTAGCCATAAAGTGATAACCTTTAATCATTGTCTTTATTAATACAACCAACTATCAGGAGAGACAACTTAAAGAGACTTAAAAg                                                                                                                                                                    <  1:358027/300‑1 (MQ=255)`  
                                                                                                                                        `tACCTTGAGTGTCTCTCTGTGTCCCTATCTGTTACAGTCTCCTAAAGTATCCTCCTAAAGTCACCTCCCAACGTCCATCCTAAAGCCAACACCTAAAGCCAACACCTAAAGACCCATCAAGTCAACGCCTATCTTAAAGTTTAAACATAAAGACCAGACCTAAAGACCAGACCTAAAAACACTACATAAAGACCAGACCTAAAGACGCCTTGTTGTTAGCCATAACGTGATAACCTTTAATCATTGTCTTTATTAATACAACTCACTATAAGGAGAGACAACTTAAAGAAACTTAAAAGAt                                                                                                                                                                  >  1:230212/1‑301 (MQ=255)`  
                                                                                                                                         `aCCTTGAGTGTCTCTCTGTGTCCCTATCTGTTACAGTCTCCTAAAGTATCCTCCTAAAGTCACCTCCTAACGTCCATCCTAAAGCCAACACCTAAAGCCTACACCTAAAGACCCATCAAGTCAACGCCTATCTTAAAGTTTAAACATAAAGACCAGACCTAAAGACCAGACCTAAAGACACTACATAAAGACCAGACCTAAAGACGCCTTGTTGTTAGCCATAAAGTGATAACCTTTAATCATTGTCTTTATTAATACAACTCACTATAAGGAGAGACAACTTAAcgactgtctcttatac                                                                                                                                                                 >  1:273743/1‑288 (MQ=255)`  
                                                                                                                                          `ccTTGAGTGTCTCTCTGTGTCCCTATCTGTTACAGTCTCCTAAAGTATCCTCCTAAATTCACCTCCTAACGTCCCTCCTCACTCCTACACCTAAAGCCTTCACCTCAAGACCCCTCTTTTCAACGCCTTTCTTTAAGTTTATTCTTATAGACCAGTCCTTCTGACCATACCTTATTACCCTTCATACTCTCCAGACCTTAATTCTCCTTGTTTTTATCCATTATGTTTTATCCTTTAATCATTTTCTTTTTTACTCCAACTCACTCTACGTAGATACACCTTactgactgtctcttttcct                                                                                                                                                                >  1:279776/1‑283 (MQ=255)`  
                                                                                                                                                                  `aTCTGTTACAGTCTCCTAAAGTATCCTCCTAAAGTCACCTCCTAACGTCCATCCTAAAGCCAACACCTAAAGCCTACACCTAAAGACCCATCAAGTCAACGCCTATCTTAAAGTTTAAACATAAATACCAGACCTAAAGACCAGACCTAAAGACACTACATAAAGACCAGACCTAAAGACGCCTTTTTGTTAGCCATAAAGTGATAACCTTTAATCATTGTCTTTATTAATACAACTCACTATAAGGAGAGACAACTTAAAGAGACTTAAAAGATTAATTTAAAATTTATCAAAAAGAGTa                                                                                                                                        >  1:423551/1‑301 (MQ=255)`  
                                                                                                                                                                   `ctcagagatgtttataagaaacaccccctAAAGTCACCTCCTAACGTCCATCCTAAAGCCAACACCTAAAGCCTAAACCTAAAGACCCAACAAGTCAACGCCTATCTTAAAGTTTAAACATAAAGCCCAGACCGAAAGACCAGACCTAAAGACACTACACAAAGACCAGACCTAAAGGCGCCTTGTTGTTATCCATAAAGTGATAACCTTTAATCATTGTCTTTATTAATACAACTCTCTATAAGGAGAGACAACTTAAAGAGACTTAAAAGATTAATTTAAAATTTATCAAAAAGAGTAt                                                                                                                                       <  1:105914/275‑1 (MQ=255)`  
                                                                                                                                                                    `cTGTTACAGTCTCCTAAAGTATCCTCCTAAAGTCACCTCCTAACGTCCATCCTAACGCGAACACCTAAAGCCTACACCTAAAGACCCATCAAGTCAACGCCTATCTTAACGTTTAAACATAAAGACCAGACCTAAAGACCAGACCTAAAGACACTACATAAAGACCAGACCTAAAGACGCCTTGTTGTTAGCCATACAGTGATAACCTTTAATCATTTTCTTTATTAATACAACTCACTATAAGGAGAAACCACATAAAGCTACTTAAAAGATTAATTTAAAATTTATCACAAAGAGTAtt                                                                                                                                      >  1:376609/1‑301 (MQ=255)`  
                                                                                                                                                                    `cTGTTACAGTCTCCTAAAGTATCCTCCTAAAGTCACCTCCTAACGTCCATCCTAAAGCCAACACCTAAAGCCTACACCTAAAGACCCATCAAGTCAACGCCTATCTTAAAGTTTAAACATAAAGACCAGACCTAAAGACCAGACCTAAAGACACTACATAAAGACCAGACCTAAAGACGCCTTGTTGTTAGCCATAAAGTGATAACCTTTAATCATTGTCTTTATTAATACAACTCACTATAAGGAGAGACAACTTAAAGAGACTTAAAAGATTAATTTAAAATTTATCAAAAAGAGTAtt                                                                                                                                      >  1:587809/1‑301 (MQ=255)`  
                                                                                                                                                                            `gTCTCCTAAAGTATCCTCCTAAAGTCACCTCCTAACGTCCATCCTAAAGCCAACACCTAAAGCCTACACCTAAAGACCCATCAAGTCAACGCCTATCTTAAAGTTTAAACATAAAGACCAGACCTAAAGACCAGACCTAAAGACACTACATAAAGACCAGACCTAAAGACGCCTTGTTGTTAGCCATAAAGTGATAACCTTTAATCATTGTCTTTATTAATACAACTCACTATAAGGAGAGACAACTTAAAGAGACTTAAAAGATTAATTTAAAATTTATCAAAAAGAGTATTGACTTaaa                                                                                                                              >  1:546280/1‑301 (MQ=255)`  
                                                                                                                                                                             `tctcCTAAAGTATCCTCCTAAAGTCACCACCTAACGTCCATCCTAAAGCCAACACCTAAAGCCTACACCTAAAGACCCATCAAGTCAACGCCTATCTTAAAGTTTAAACATAAAGACCAGACCTAAAGACCAGACCTAAAGACACTACATAAAGACCAGACCTAAAGACGCCTTGTTGTTAGCCATAAAGTGATAACCTTTAATCATTGTCTTTATTAATACAACTCACTATAAGGAGAGACAACTTAAAGAGACTTAAAAGATTAATTTAAAATTTATCAAAAAGAGTATTGACTTAAAg                                                                                                                             <  1:225332/301‑1 (MQ=255)`  
                                                                                                                                                                              `ttgtataaaaaacagccaaaaGTCACCTCCTAACGTCCAGCCTAAAGCCAACACCTAAAGCCTACACCTAAAGACCCATCAAGTCAACGACTATCTTAAAGTTTAAACATAAAGACCAGACCTAAAGACCAGACCTAAAGACACTACATAAAGACCAGACCTAAAGACGCCTTGTTGTTAGCCATAAAGTGATAACCTTTAATCATTGTCTTTATTAATACAACTCACTATAAGGAGAGACAAATTAAAGAGACTTAAAAGATTAATTTAAAATTTATCAAAAAGAGTATTGACTTAAAGt                                                                                                                            <  1:619136/283‑1 (MQ=255)`  
                                                                                                                                                                              `ctcCTAAAGTATCCTCCTAAAGTCACCTCCTAACGTCCATCCTAAAGCCAACACCTAAAGCCTACACCTAAAGACCCATCAAGTCAACGCCTATCTTAAAGTTTAAACATAAAGACCAGACCTACAGACCAGACCTAAAGACACTACGTACAGACCAGACCTAAAGACGCCTTGTTGTTAGCCATAACGTGATAACCTTTAATCATTGTCTTTATTAATACAACTACCTATAAGGCGAGACACCTTAAAGAGACTTAAAATATTAATTAAAAATTTATCAAAAACAGTATTCACTTAAAGt                                                                                                                            >  1:411671/1‑301 (MQ=255)`  
                                                                                                                                                                               `gggctcggagatgtgtataagagacagcctAACGTCCAGCCTAAAGCCAACACCTAAAGCCTACACCTAAAGACCCATCAAGTCAACGCCTATCTTAAAGTTTAAACATAAAGACCAGACCTAAAGACCAGACCTAAAGACACTACATAAAGACCAGACCTAAAGACGCCTTGTTGTTAGCCATAAAGTGATAACCTTTAATCATTGTCTTTATTAATACAACTCACTATAAGGAGAGACAACTTAAAGAGACTTAAAAGATTAATTTAAAATTTATCAAAAAGAGTATTGACTTAAAGTc                                                                                                                           <  1:129634/274‑1 (MQ=255)`  
                                                                                                                                                                                `ccTAAAGTATCCTCCTTAAGTCACCTCCTAACGTCCATCCTAAAGCCAACACCTAAAGCCTACACCTAAAGACCCATCAAGTCAACGCCTATCTTAAAGTTTAAACATAAAGACCAGACCTAAAGACCAGACCTAAAGACACTACATAAAGACCAGACCTAAAGACGCCTTGTTGTTAGCCATAAAGTGATAACCTTTAATCATTGTCTTTATTAATACAACTCACTATAAGGAGAGACAACTTAAAGAGACTTAAAAGATTAATTTAAAATTTATCAAAAAGAGTATTGACTTAAAGTCt                                                                                                                          >  1:513202/1‑301 (MQ=255)`  
                                                                                                                                                                                      `gTATCCTCCTAAAGTCACCTCCTAACGTCCATCCTAAAGCCAACACCAAAAGCCTACACCTACAGACCCATCAAGTCAACGCCTATCTTAAAGTTTAAACATAAAGACCAGACCTAAAGACCAGACCTAAAGACACTACATAAAGACCAGACCTAAAGACGCCTTGTTGTTAGCCATAAAGTGATAACCTTTAATCATTGTCTTTATTAATACAACTCACTATAATGAGCGACAACTTAAAGAGACTTAAAAGATTAATTTAAAATTTATCAAAAAGAGTATTGACTTAAAGTCTAACCta                                                                                                                    >  1:3461/1‑301 (MQ=255)`  
                                                                                                                                                                                           `ctcctAAAGTCACCTCCTAACGTCCATCCTAAAGCCAACACCTAAAGCCTACACCTAAAGACCCATCAAGTCAACGCCTATCTTAAAGTTTAAACATAAAGACCAGACCTACAGACCAGACCTAAAGACACTACATAAAGACCAGACCTAAAGACGCCTTGTTGTTAGCCATAAAGTGATAACCTTTAATCATTGTCTTTATTAATACAACTCACTATAAGGAGAGACAACTTAAAGAGACTTAAAAGATTAATTTAAAATTTATCAAAAAGAGTATTGACTTAAAGTCTAACCTCTAGGa                                                                                                               >  1:534478/1‑301 (MQ=255)`  
                                                                                                                                                                                                `aaaGTCACCTCCTAACGTACATCCTAAAGCCAACTACTAAAGACTAAACCTAAAGACCCATCAAGTCACCGCCTATCTTAAAGTTTAAACAT‑AAGACCAGACCTAAAGACCAGACCTAAAGACACTACATAAAGACCAGTCCTAAAGACGCCTTGTTGTTAGCCATAAAGTGATAACCTTTAATCATTGTCTTTATTAATACAACTCACTATAAGGAGAGACAACTTAAAGAGACTTAAAAGATTAATTTAAAATTTATCAAAAAGAGTATTGACTTAAAGTCTAACCTATAGGATACTTa                                                                                                         <  1:507458/301‑1 (MQ=255)`  
                                                                                                                                                                                                      `gcctcctAACGTCAATCATAAAGACAACACCTAAAGACTAAACATAAAGACCCATCAAATCAAAGCCTATCTTAAAGTTTAAACATAAAGACCAGACCTAAAGACCAGACCTAAAGAAACTACATAAAGACCAGACCTAAAGAAGCCTTGTTGTTAGCCATAAAGTGATAACCTTTAATCATAGTCTTTATTAATACAACTCACTATAAGGAGAGACAACTTAAAGAGACTTAAAAGATTAATTTAAAATTTATCAAAAAGAGTATTGACTTAAAGTCTAACCTATAGGATACTTACAGcc                                                                                                    <  1:166586/300‑1 (MQ=255)`  
                                                                                                                                                                                                      `aCCTCCTAACGTCCATCCTAAAGCCAACACCTAAAGCCTACACCTAAAGACCCATCAAGTCAACGCCTATCTTAAAGTGTAAACATAAAGACCAGACCTAAAGACCAGACCTAAAGACACTACATAAAGACCAGACCTAAAGACGCCTTGTTGTTAGCCATAAAGTGATAACCTTTAATCATTGTCTTTATTAATACAACTCACTATAAGGAGAGACAACTTAAAGAGACTTAAAAGATTAATTTAAAATTTATCAAAAAGAGTATTGACTTAAAGTCTAACCTATAGGATACTTACAGcc                                                                                                    <  1:646228/301‑1 (MQ=255)`  
                                                                                                                                                                                                          `cctAACGTCCATCCTAAAGCCAACACCTAAAGCCTACACCTAAAGACCCATCAAGTCAACGCCTATCTTAAAGTTTAAACATAAAGACCAGACCTAAAGACCAGACCTAAAGACACTACATAAAGACCAGACCTAAAGACGCCTTGTTGTTATCCATAAAGTGATAACCTTTAATCATTGTCTTTATTAATACAACTCACTATAAGGAGAGACAACTTAAAGAGACTTAAAAGATTAATTTAAAATTTATCAAAAAGAGTATTGACTTAAAGTCTAACCTATAGGATACTTACAGCCATCg                                                                                                >  1:395518/1‑301 (MQ=255)`  
                                                                                                                                                                                                          `cctAACGTCCATCCTAAAGCCAACACCTAAAGCCTACACCTAAAGACCCATCAAGTCAACGCCTATCTTAAAGTTTAAACATAAAGACCAGACCTAAAGACCAGACCTAAAGACACTACATAAAGACCAGACCTAAAGACGCCTTGTTGTTAGCCATAAAGTGATAACCTTTAATCATTGTCTTTATTAATACAACTCACTATAAGGAGAGACAACTTAAAGATACTTAAAAGATTAATTTAAAATTTATCAAAAAGAGTATTGACTTAAAGTcctgtctcttatacacatctccgagccc                                                                                                >  1:502338/1‑274 (MQ=255)`  
                                                                                                                                                                                                          `cctAACGTCCATCCTAAAGCCAACACCT‑AAGCCCTCAACTTAAGACCCATCAAGTCAACGCCCAACCTAAAATTTAAACCTAAAGACCAAACCTAAAGAACAGACCTAAAGACAATACATAAAGACCAAACCTAAAGACGCCGTGTTTTTAGCCATAAAGTGATAACCTTTAATCATTGTCCTTATTTATACAACTCACTATAAGGAGAAAAAACTTTAAGAAACTTAAAAAATTAAATTAAAATTTAATAAAAAGAGTAATTAATTTAAGTCTAACCCAATAGATACTTACAGcctgtat                                                                                               >  1:640005/1‑296 (MQ=255)`  
                                                                                                                                                                                                              `aCGTCCAACCTAAAGCCAACACCTAAAGCCTACACCCAAAGACCCATCAAGTCAACGCCTATCTTAAAGTTTAAACATAAAGACCAGACCTAAAGACCAGACCTAAAGACACTACATAAAGACCAGACCTAAAGACGCCTTGTTGTTAGCCATAAAGTGATAACCTTTAATCATTGTCTTTATTAATACAACTCACTATAAGGAGAGACAACTTAAAGAGACTTAAAAGATTAATTTAAAATTTATCAAAAAGAGTATTGACTTAAAGTCTAACCTATAGGATACTTACAGCCATCAagag                                                                                            <  1:495963/301‑1 (MQ=255)`  
                                                                                                                                                                                                                `aacagtacTAAAGCCAACACCTAAAGCCGACACCTAAAGACCCATCAAGTCAACGCCTATCTTAAAGCTTAAACATAAAGACCAGACCTAAAGACCAGACCTAAAGACACTATATAAAGACCACACCTAAAGACGCCTTGTTGTTAGACATAAAGTGATAACCTTTAATCATTGTCTTTATTAATACTACTCACTATAAGGAGAGACAACTTAAAGAGACTTAAAAGATTAATTTAAAATTTATCAAAAAGAGTATTGACTTAAAGTCTAACCTATAGGATACTTACAGCCATCGAGAggg                                                                                          <  1:406018/294‑1 (MQ=255)`  
                                                                                                                                                                                                                     `tCCTAAAGCCAACACCTAAAGCCTACACCTAAAGACCCATCAAGTCAACGCCTATCTTAAAGTTTAAACATAAAGACCCGACCTAAAGACCAGACCTAAAGACACTACATAAAGACCAGACCTAAAGACGCCTTGTTGTTAGCCATAAAGTGATAACCTTTAATCATTGTCTTTATTAATACAACTCACTATAAGGAGAGACAACTTAAAGAGACTTAAAAGATTAATTTAAAATTTATCAAAAAGAGTATTGACTTAAAGTCTAACATATAGGATACTTACAGCAATCGAGAGGAACACg                                                                                     >  1:63162/1‑301 (MQ=255)`  
                                                                                                                                                                                                                              `cAACACCTAAAGCCGACACCTAAAGACCCATCAAGTCAACGCCTATCTTAAAGTTTAAACATAAAGACCAGACCTAAAGACCAGACCTAAAGACACTACATAAAGACCAGCCCTAAAGACGCCTTGTTGTTAGCCATAAAGTGATAACCTTTAATCATTGTCTTTATTAATACAACTCACTATAAGGAGAGACAACTTAAAGAGACTTAAAAGATTAATTTAAAATTTATCAAAAAGAGTATTGACTTAAAGTCTAACCTATAGGATACTTACAGCCATCGAGAGGGACACGGCGAATAGc                                                                            <  1:268197/301‑1 (MQ=255)`  
                                                                                                                                                                                                                                  `acCTAAAGCCTACACCTAAAGACCCATCAAGTCAACGCCTATCTTAAAGTTTAAACATAAAGACCAGACCTAAAGACCAGACCTAAAGACACTACATAAAGACCAGACCTAAAGACGCCTTGTAGTAAGCCATAAAGTGATAACCTTTAATCATTGTCTTTATTAATACAACTCACTATAAGGAGACACAACTTAAAGAAAATTAAAAGATTAATTTAAAATTTATCAAAAAGAATATGGACTTAAGTGCTAACCTATAGGATACTTACAACCATCGAGATGAACACGGCAAATACCCATc                                                                        >  1:81219/1‑301 (MQ=255)`  
                                                                                                                                                                                                                                   `ccTAAAGCCTACACCTAAAGACCCATCAAGTCAACGCCTATCTTAAAGTTTAAACATAAAGACCAGACCTAAAGACCAGACCTAAAGACACTACATAAAGACCAGACCTAAAGACGCCTTGTTGTTAGCCATAAAGTGATCACCTTTAATCATTGTCTTTATTAATACAACTCACTATAAGGAGAGACAACTTAAAGAGACTTAAAAGATTAATTTAAAATTTATCAAAAAGAGTATTGACTTAAAGTCTAACCTATAGGATACTTACCGCCATCGAGAGGAACACGGCGAATAGCCATcc                                                                       >  1:625256/1‑301 (MQ=255)`  
                                                                                                                                                                                                                                   `ccTAAAGCCTACACCTAAAGACCCATCAAGTCAACGCCTATCTTAAAGTTTAAACATAAAGACCAGACCTAAAGACCAGACCTAAAGACACTACATAAAGACCAGACCTAAAGACGCCTTGTTGTTAGCCATAAAGTGATAACCTTTAATCATTGTCTTTATTAATACAACTCACTATAAGGAGAGACAACTTAAAGAGACTTAAAAGATTAATTTAAAATTTATCAAAAAGAGTATTGACTTAAAGTCTAACCTATAGGATACTTACAGCCATCGAGAGGGACACGGcctgtctcttata                                                                       >  1:235546/1‑289 (MQ=255)`  
                                                                                                                                                                                                                                   `ccTAAAGCCTACACCTAAAGACCCATCAAGTCAACGCCTATCTTAAAGTTTAAACATAAAGACCAGACCTAAAGACCAGACCTAAAGACACTACATAAAGACCAGACCTAAAGACGCCTTGTTGTTAGCCATAAAGTGA‑AACCTTTAATCATTGTCTTTATTAATACAACTCACTATAAGGAGAGACAACTTAAAGAGACTTAAAAGATTAATTTAAAATTTATCAAAAAGAGTATTGACTTAAAGTCTAACCTATAGGATACTTACAGCCATCGAGAGGGACACGGCGAATAGCCATccc                                                                      >  1:5648/1‑301 (MQ=255)`  
                                                                                                                                                                                                                                        `aGCCTACACCTAAAGACCCATCAAGTCAACGCCTATCTTAAAGTTTAAACATAAAGACCAGACCTAAAGACCAGACCTAAAGACACTACATAAAGACCAGACCTAAAGACGCCTTGTTGTTAGCCATAAAGTGATAACCTTTAATCATTGTCTTTATTAATACAACTCACTATAAGGAGAGACAACTTAAAGAGACTTAAAAGATTAATTTAAAATTTATCAAAAAGAGTATTGACTTAAAGTCTAACCTATAGGATACTTACAGCCATCgctgtctcttatacacatctccgagcccacg                                                                  >  1:44938/1‑271 (MQ=255)`  
                                                                                                                                                                                                                                              `cacCTAAAGACCCATCAAGTCAACGCCTATCTTAAAGTTTAAACATAAAGACCAGACCTAAAGACCAGACCTAAAGACACTACATAAAGACCAGACCTAAAGACGCCTTGTTGTTAGCCATAAAGTGATAACCTTTAATCATTGTCTTTATTAATACAACTCACTATAAGGAGAGACAACTTAAAGAGACTTAAAAGATTAATTTAAAATTTATCAAAAAGAGTATTGACTTAAAGTCTAACCTATAGGATACTTACAGCCATCGAGAGGGACACGGCGAATAGCCATCCCAATCGACAcc                                                            >  1:418119/1‑301 (MQ=255)`  
                                                                                                                                                                                                                                                `ccTAAAGACCCATCAAGTCAACGCCTATCTTAAAGTTTAAACATAAAGACCAGACCTAAAGACCAGACCTAAAGACACTACATAAAGACCAGACCTAAAGACGCCTTGTTGTTAGCCATAAAGTGATAACCTTTAATCATTGTCTTTATTAATACAACTCACTATAAGGAGAGACAACTTAAAGAGACTTAAAAGATTAATTTAAAATTTATCAAAAAGAGTATTGACTTAAAGTCTAACCTATAGGATACTTACAGCCATCGAGAGGGACACGGCGAATAGcctgtctcttctacacatc                                                          >  1:308668/1‑284 (MQ=255)`  
                                                                                                                                                                                                                                                          `cATCAAGTCAACGCCTATCTTAAAGTTTAAACATAAAGACCAGACCTAAAGACCAGACCTAAAGACACTACATAAAGTCCAGACCTAAAGACGCCTTGTTGTTAGCCATAAAGTGATAACCTTTAATCATTGTCTTTATTAATACAACTCACTATAAGGAGAGACAACTTAAAGAGACTTAAAAGATTAATTTAAAATTTATCAAAAAGAGTATTGACTTAAAGTCTAACCTATAGGATACTTACAGCCATCGAGAGGGACACGGCGAATAGCCATCCCCATCGACACCGGGTTCAACCgg                                                >  1:442832/1‑301 (MQ=255)`  
                                                                                                                                                                                                                                                               `aGTCAACGCCTATCTTAAAGTTTAAACATTAAGACCAGACCTAAAGACCAGACCTAAAGACACTACATAAAGACCAGACCTAAAGACGCCTTGTTGTTAGCCATAAAGTGATAACCTTTAATCATTGTCTTTATTAATACAACTCACTATAAGGAGAGACAACTTAAAGAGACTTAAAAGATTAATTTAAAATTTATCAAAAAGAGTATTGACTTAAAGTCTAACCTATAGGATACTTACAGCCATCGAGAGGGACACGGCGAATAGCCATCCCAATCGACACCGGGGTCAACCGGATAAg                                           <  1:160009/301‑1 (MQ=255)`  
                                                                                                                                                                                                                                                               `aGTCAACGCCTATCTTAAAGTTTAAACATAAAGACCAGACCTAAAGACCAGACCTAAATACACTACATAAAGACCAGACCTAAAGACGCCTTGTTGTTAGCCATAAAGTGATAACCTTTAATCATTGTCTTTATTAATACAACTCACTATAAGGAGAGACAACTTAAAGAGACTTAAAAGATTAATTTACAATTTATCAAAAAGAGTATTGACTTAAAGTCTAACCTATAGGATACTTACAGCCATCGAGAGGGACACGGCGAATAGCCATCCCAATCGACACCGGGGTCAACCGGATAAg                                           >  1:364641/1‑301 (MQ=255)`  
                                                                                                                                                                                                                                                                  `aaaCGCCTATCTTAAAGTTTAAACATAAAGACCAGACCTAAAGACCAGACCTAAAGACACTACATAAAGACCAGACCGAAAGACGTCTTGTTGTTAGCCATAAAGTGATAACCTTTAATCATTGTCTTTATTAATACAACTCACTATAAGGAGAGACAACTTAAAGAGACTTAAAAGATTAATTTAAAATTTATCAAAAAGAGTATTGACTTAAAGTCTAACCTATAGGATACTTACAGCCATCGAGAGGGACACGGCGAATAGCCATCCCAATCGACACCGGGGTCAACCGGATAAGTAg                                        <  1:460308/300‑1 (MQ=255)`  
                                                                                                                                                                                                                                                                    `aCGCCTATCTTAAAGTTTAAACATAAAGACCAGACCTAAAGACCAGACCGAAAGACAATACATAAAGACCAAACCTAAAGACGCCGTGTTGTTAGCCATAAAGTGATAACCTTTAATCATTGTCTTTATTAATACAAATCACTATAAGGAGAGACAACTTAAAGAGACTTAAAAGATGAATATAAAATTTATCAAAAAGAGTATTGACTTAAAGTCTAACCTATAGGATACTTACAGCCATCGAGAGGGACACGGCGAATAGCCATCCCAATCGACACCGGGGTCAACCGGATAAGTAGAc                                      <  1:147967/301‑1 (MQ=255)`  
                                                                                                                                                                                                                                                                                               `gaccagacctaaagaccagacctaaaGACACTACATAAAGACCAGACCTAAAGACGCCTTGTTGTTAGCCATAAAGTGATAACCTTTAATCATTGTCTTTATTAATACAACTCACTATAAGGAGAGACAACTTAAAGAGACTTAAAAGATTAATTTAAAATTTATCAAAAAGAGTATTGACTTAAAGTCTAACCTATAGGATACTTACAGCCATCGAGAGGGACACGGCGAATAGCCATCCCAATCGACACCGGGGTCAACCGGATAAGTAGACAGCCTGATAAGTCGCACGaactgtctc           >  1:171893/1‑294 (MQ=255)`  
                                                                                                                                                                                                                                                                                                      `cctaaagaccagacctaaagcaACTACATAAAGACCAGACCTAAAGACGCCTTGTTGTTAGCCATAAAGTGATAACCTTTAATCATTGTCTTTATTAATACAACTCACTATAAGGAGAGACAACTTAAAGAGACTTAAAAGATTAATTTAAAATTTATCAAAAAGAGTATTGACTTAAAGTCTAACCTATAGTATAATTACAGCCATCGAGAGGGACACGGCGAATAGCCATCCCAATCGACACCGGGGTCAACCGGATAAGTAGACAGCCTGATAAGTCGCACGAAAAACAGGTATTGac    <  1:388210/301‑1 (MQ=255)`  
                                                                                                                                                                                                                                                                                                      `cctaaagaccagacctaaagacACTACATAAAGACCAGACCTAAAGACGCCTTGTTGTTAGCCATAAAGTGATAACCTTTAATCATTGTCTTTATTAATACAACTCACTATAAGGAGAGACAACTTAAAGAGACTTAAAAGATTAATTTAAAATTTATCAAAAAGAGTATTGACTTAAAGTCTAACCTATAGGATACTTACAGCCATCGAGAGGGGCACGGCGAATAGCCATCCCAATCGACACCGGGGTCAACCGGATAAGTGGACAGCCTGATAAGTCGCACGAAAAACAGGTATTGac    <  1:539487/301‑1 (MQ=255)`  
                                                                                                                                                                                                                                                                                                       `ctaaagaccagacctaaagacACTACATAAAGACCAGACCTAAAGACGCCTTGTTGTTAGCCATAAAGTGATAACCTTTAATCATTGTCTTTATTAATACAACTCACTATAAGGAGAGACAACTTAAAGAGACTTAAAAGATTAATTTAAAATTTATCAAAAAGAGTATTGACTTAAAGTCTAACCTATAGGATACTTACAGCCATCGAGAGGGACACGGCGAATAGCCATCCCAATCGACACCGGGGTCAACCGGATAAGTAGACAGCCTGATAAGTCGCACGAAAAACAGGTATTGaca   <  1:7610/301‑1 (MQ=255)`  

                                                                                                                                                                                                                                                                                                        `|`                                                                                                                                                                                                                                                                                                              
`‑‑TCTCACAGTGTACGGACCTAAAGTTCCCCCATAGGGGGTACCTAAAGCCCAGCCAATCACCTAAAGTCAACCTTCGGTTGACCTTGAGGGTTCCCTAAGGGTTGGGGATGACCCTTGGGTTTGTCTTTGGGTGTTACCTTGAGTGTCTCTCTGTGTCCCTATCTGTTACAGTCTCCTAAAGTATCCTCCTAAAGTCACCTCCTAACGTCCATCCTAAAGCCAACACCTAAAGCCTACACCTAAAGACCCATCAAGTCAACGCCTATCTTAAAGTTTAAACATAAAGACCAGACCTAAAGACCAGACCTAAAGACACTACATAAAGACCAGACCTAAAGACGCCTTGTTGTTAGCCATAAAGTGATAACCTTTAATCATTGTCTTTATTAATACAACTCACTATAAGGAGAGACAACTTAAAGAGACTTAAAAGATTAATTTAAAATTTATCAAAAAGAGTATTGACTTAAAGTCTAACCTATAGGATACTTACAGCCATCGAGAGGGACACGGCGAATAGCCATCCCAATCGACACCGGGGTCAACCGGATAAGTAGACAGCCTGATAAGTCGCACGAAAAACAGGTATTGACAA  >  NC_001604.1/1‑595`

  

Alignment Legend

Aligned base mismatch/match (shaded by quality score): `ATCG/ATCG` `< 3 ≤` `ATCG/ATCG` `< 10 ≤` `ATCG/ATCG` `< 20 ≤` `ATCG/ATCG` `< 34 ≤` `ATCG/ATCG` `< 38 ≤` `ATCG/ATCG`

Unaligned base: `atcg`    Masked matching base: `atcg`    Alignment gap: `‑`    Deleted base: `‑`

Reads not counted as support for junction

read\_name Not counted due to insufficient overlap past the breakpoint.

read\_name Not counted due to not crossing MOB target site duplication.
